# Prevalence of antibiotic resistance in multi-drug resistant coagulase-negative staphylococci isolated from invasive infection in very low birth weight neonates in two Polish NICUs

**DOI:** 10.1186/1476-0711-12-41

**Published:** 2013-12-20

**Authors:** Monika Brzychczy-Wloch, Maria Borszewska-Kornacka, Ewa Gulczynska, Jadwiga Wojkowska-Mach, Malgorzata Sulik, Monika Grzebyk, Malgorzata Luchter, Piotr B Heczko, Malgorzata Bulanda

**Affiliations:** 1 Department of Bacteriology, Microbial Ecology and Parasitology, Chair of Microbiology, Jagiellonian University Medical College, Czysta Street 18, 31-121, Krakow, Poland; 2 Neonatal and Intensive Care Department Medical University of Warsaw, Warsaw, Poland; 3 Polish Mother’s Memorial Hospital, Lodz, Poland; 4 Duchess Anna Mazowiecka Teaching Hospital, Warsaw, Poland; 5 Department of Epidemiology of Infection, Chair of Microbiology, Jagiellonian University Medical College, Krakow, Poland

**Keywords:** Multi-drug resistant coagulase-negative staphylococci, Resistance genes, Very-low-birth-weight neonates, Nosocomial infections

## Abstract

**Background:**

Multi-drug resistant coagulaso-negative staphylococci (CNS) have become an increasing problem in nosocomial infections connected with the presence of medical devices. The paper aimed to analyze the prevalence of antibiotic resistance in CNS isolated from invasive infection in very low birth weight (VLBW) neonates.

**Methods:**

Continuous prospective target surveillance of infections was conducted in 2009 at two Polish NICUs that participated in the Polish Neonatology Surveillance Network (PNSN). The study covered 386 neonates with VLBW (≤1500 g), among which 262 cases of invasive infection were detected with predominance of CNS (123; 47%). Altogether, 100 CNS strains were analyzed. The resistance phenotypes were determined according to EUCAST. Resistance genes: *mecA, ermA, ermB, ermC, msrA, aac(6')/aph(2''), ant(4')-Ia* and *aph(3')-IIIa* were detected using multiplex PCR.

**Results:**

The most common species was *S. epidermidis* (63%), then *S. haemolyticus* (28%) and other CNS (9%). Among *S. epidermidis*, 98% of isolates were resistant to methicillin, 90% to erythromycin, 39% to clindamycin, 95% to gentamicin, 60% to amikacin, 36% to ofloxacin, 2% to tigecycline, 3% to linezolid and 13% to teicoplanin. Among *S. haemolyticus* isolates, 100% were resistant to methicillin, erythromycin and gentamicin, 18% to clindamycin, 50% to amikacin, 86% to ofloxacin, 14% to tigecycline and 4% to teicoplanin. No resistance to linezolid was detected for *S. haemolyticus* isolates. Moreover, all isolates of *S. epidermidis* and *S. haemolyticus* were susceptible to vancomycin. The *mecA* gene was detected in 98% of *S. epidermidis* isolates and all of *S. haemolyticus* ones. Among macrolide resistance isolates, the *ermC* was most common in *S. epidermidis* (60%) while *msrA* was prevalent in *S. haemolyticus* (93%). The *ermC* gene was indicated in all isolates with cMLS_B_, whereas *mrsA* was found in isolates with MS_B_ phenotype. Of the aminoglycoside resistance genes, *aac(6')/aph(2'')* were present alone in 83% of *S. epidermidis,* whereas *aac(6')/aph(2''*) with *aph(3')-IIIa* were predominant in 84% of *S. haemolyticus*.

**Conclusions:**

Knowing the epidemiology and antibiotic resistance of CNS isolated from invasive infection in VLBW neonates is a key step in developing targeted prevention strategies and reducing antibiotic consumption.

## Introduction

Coagulase-negative staphylococci (CNS) rank among opportunistic pathogens being a frequent etiologic agent of nosocomial infections connected with the presence of medical devices. The cause for this should be sought in the ability of CNS to create biofilm whereby they pose a particular threat for people with valve prostheses and the ones with implants or catheters [1]. A group which is particularly susceptible to hospital infections are very low birth weight (VLBW) neonates in which CNS are identified as 48 up to 80% of all etiologic agents causing late-onset diseases (LOD). The most frequently isolated species are *Staphylococcus epidermidis* (58% – 76%) and then *Staphylococcus haemolyticus* (14% – 32%) [2-4].

Medicating infections in neonates poses a big problem as there is the need to use a therapy quickly. In the event of infections caused by hospital pathogens, the empirical treatment is adjusted based on information on drug-resistance of the bacteria persisting in the ward. A high percentage of *S. epidermidis* and *S. haemolyticus* isolates coming from neonates are resistant to many antibiotics: methicillin (86% – 100%), gentamicin (80% – 100%), erythromycin (65% – 100%), oxacillin (92% – 100%), or clindamycin (80% – 100%) [5-7].

The growing importance of coagulase-negative staphylococci, including multidrug-resistant strains, among other etiologic agents of nosocomial infections, forcing researchers to look for the most effective ways to combat these pathogens. Therefore, the aim of the project was to analyze the prevalence of antibiotic resistance in invasive coagulase-negative staphylococci isolates derived from very low birth weight neonates hospitalized in two Polish Neonatal Intensive Care Units (NICUs).

## Methods

Continuous prospective target surveillance of infections was conducted from 1.01.2009 to 31.12.2009 at two Polish NICUs that participated in the Polish Neonatology Surveillance Network (PNSN). The surveillance concerned infants hospitalized at cooperating units whose birth weight was ≤1500 grams (from birth to discharge, or until the weight of 1800 grams or death). All cases of infections were subject to registration, regardless of the time of occurrence of the first symptoms as early-onset infection (EOI) or late-onset infection (LOI). Case patients were defined according to Gastmeier et al. [8] with modifications as neonates with very low birth weight (VLBW) when they had clinical signs of septicemia or of pneumonia, as previously described [9]. The study covered 386 VLBW neonates, among which 262 cases of LOI infection were detected with predominance of CNS (123; 47%) including blood stream infections (54 cases, 43.9%), pneumonia (58 cases, 47.2%) and others.

(11 cases, 8.9%). Altogether, one hundred invasive coagulase-negative staphylococci isolates were collected and stored at the temperature of -80°C. Preliminary identification of species was performed using API Staph (bioMerieux) and than multiplex PCR method according to Pereira et al. [10].

### Antibiotic susceptibility testing

To determine the drug-resistance phenotype, the Kirby-Bauer disk diffusion method was used in which Müller-Hinton 2 LAB-AGAR™ (Biocorp) and antibiotic disks (Oxoid) were utilized: cefoxitin 30 μg, clindamycin 2 μg, erythromycin 15 μg, tigecycline 15 μg, ofloxacin 5 μg, gentamicin 10 μg, amikacin 30 μg, linezolid 10 μg and the E-test method enabling determination of MIC (Minimal Inhibitory Concentration) for teicoplanin and vancomycin (bioMerieux). The results were interpreted according to EUCAST (The European Committee on Antimicrobial Susceptibility Testing) 2012 [10].

### Polymerase chain reaction, PCR

To isolate DNA, the Genomic Mini Set (A&A Biotechnology) was used according to the manufacturer’s protocol. The presence of species-specific genes to *S. haemolyticus* or *S. epidermidis* and methicillin-resistance *mec*A gene was confirmed using multiplex PCR amplification according to Pereira et al. [11] with specific primers (Genomed). To detect genes coding the erythromycin-resistance, the multiplex PCR reaction was conducted on *ermA, ermC, msrA* genes and PCR on the *ermB* gene, according to the procedure described by Zmantar et al. [12]. The determination of *aac(6′)/aph(2″*), *aph(3′)-IIIa* and *ant(4′)-Ia* genes coding the aminoglycoside-resistance was conducted according to the procedure by Choi et al. [13]. The final pictures from electrophoresis were processed using QuantityOne software, as well as GelDoc2000 device (Bio-Rad, USA).

## Results

**Figure 1:**
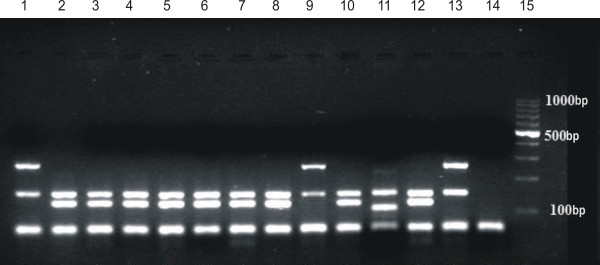
**An example of the outcome of the multiplex PCR reaction for determining the species of *Staphylococcus aureus *(108 bp), *Staphylococcus epidermidis *(124 bp), *Staphylococcus haemolyticus *(271 bp) and for detecting the presence of *mecA *gene (154 bp).** Legend: Lanes 1 – 10 studied samples; 11 – positive control reference *S. aureus* ATCC 33592; 2 – positive control reference *S. epidermidis* ATCC 700296; 13 – positive control reference *S. haemolyticus* ATCC 29970, 14 – negative control; 15 – marker (Eurx Perfect TM 100 bp DNA Ladder).

The species identification with the multiplex PCR indicated that 63% of the tested CNS isolates belonged to *S. epidermidis* species, while 28% to *S. haemolyticus* (Figure 1)*.* The remaining 9% (n = 9) of isolates belonged to other CNS species, including 4% (n = 4) *S. warneri*, 2% (n = 2) *S. hominis*, 2% (n = 2) *S. xylosus* and 1% (n = 1) *S. capitis.*

**Figure 2:**
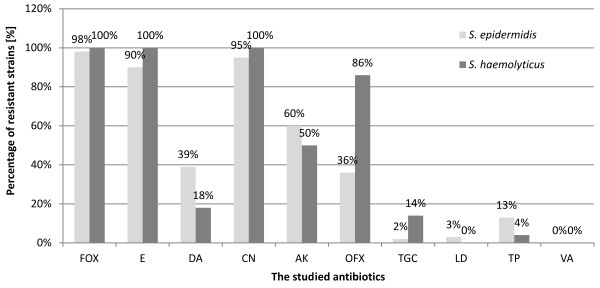
**The percentage of isolates of the species *S. epidermidis *** **and S** ***. haemolyticus*, resistant to the studied antibiotics.**

A detailed analysis of drug resistance with the use of phenotypic and genotypic methods was carried out for isolates of the species *S. epidermidis* (n = 63) and *S. haemolyticus* (n = 28). Among the isolates tested, there was a very high percentage of multi-drug resistant strains. Among *S. epidermidis*, 98% (n = 62) of isolates were resistant to methicillin, 90% (n = 57) to erythromycin, 39% (n = 25) to clindamycin, 95% (n = 60) to gentamicin, 60% (n = 38) to amikacin, 36% (n = 23) to ofloxacin, 2% (n = 1) to tigecycline, 3% (n = 2) to linezolid and 13% (n = 8) to teicoplanin. Among *S. haemolyticus* isolates, 100% (n = 28) were resistant to methicillin, erythromycin and gentamicin, 18% (n = 5) to clindamycin, 50% (n = 14) to amikacin, 86% (n = 24) to ofloxacin, 14% (n = 4) to tigecycline and 4% (n = 1) to teicoplanin. No resistance to linezolid was detected for *S. haemolyticus* isolates. Moreover, all isolates of *S. epidermidis* and *S. haemolyticus* were susceptible to vancomycin (Figure 2).

Using E-test, the values for MIC_50_ and MIC_90_ were determined for teicoplanin and vancomycin. For *S. epidermidis*, MIC_50_ and MIC_90_ for teicoplanin was equal to 2 and 6, respectively, while for vancomycin, it was 2 and 3. For *S. haemolyticus*, MIC_50_ and MIC_90_ for teicoplanin was equal to 2 and 3, respectively, and for vancomycin, it was 2 and 3.

**Figure 3:**
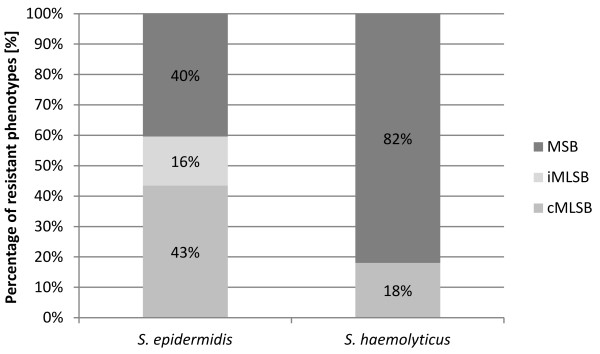
**The percentage of isolates of the species *S. epidermidis *** **and S** ***. haemolyticus *** **with macrolide resistance phenotype cMLS**_**B**_**, iMLS**_**B **_**and MS**_**B**_**.**

Among *S. epidermidis* isolates resistant to macrolides (n = 57), the cMLS_B_ and MS_B_ phenotypes were most common, and performed in 43% (n = 25) and 40% (n = 23) of isolates, respectively. While the iMLS_B_ phenotype was present in 16% (n = 9) of *S. epidermidis*. The MS_B_ phenotype was predominant among *S. haemolyticus* `isolates (n = 28), and appeared in 82% of isolates (n = 23), whereas cMLS_B_ phenotype was detected in 18% of the isolates (n = 5). In the case of *S. heamolyticus*, the MS_B_ phenotype was not detected (Figure 3).

The presence of *mecA* gene was confirmed with the multiplex PCR method in 62 *S. epidermidis* (98%) isolates and in 28 isolates of the species *S. haemolyticus* (100%) (Figure 1).

**Figure 4:**
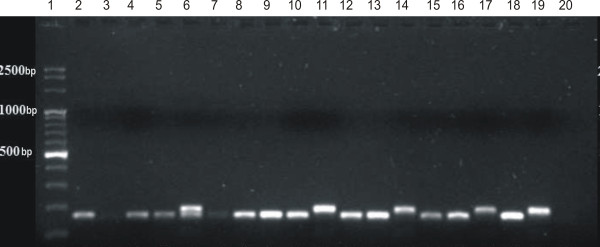
**An example of the outcome of the multiplex PCR reaction for determining the genes coding macrolide resistance *ermA *(139 bp), *ermC *(190 bp) and *msrA *(163 bp).** Legend: Lane 1 –marker (Eurx Perfect TM 100 bp DNA Ladder ); 2 – 17 studied samples; 18 – positive control reference *msrA* W2/28; 19 – positive control reference *ermC* W2/55; 20 – negative control.

The *ermC* gene was predominant in *S. epidermidis* isolates (n = 34; 60%), while in *S. haemolyticus* was present only in 2 isolates (7%)*.* On the other hand, *msrA* gene was prevalent in *S. haemolyticu*s isolates (n = 26; 93%), while in *S. epidermidis* it was much less frequent (n = 23; 40%). It is noteworthy to remark that one of the *S. haemolyticus* isolates possessed both *msrA* and *ermC* genes simultaneously. The presence of *ermA* oraz *ermB* genes was not demonstrated in the studied isolate pool (Figure 4).

*S. epidermidis* isolates resistant to macrolides, of cMLS_B_ and iMLS_B_ resistance phenotype, possessed *ermC* gene (n = 34; 60%). While five *S. haemolyticus* isolates of cMLSB resistance phenotype demonstarted the presence of various genes, including three with *msrA* gene, in the second one *ermC* and in the remaining one of *ermC* and *msrA* genes at the same time. In all *S. epidermidis* isolates (n = 23; 100%) and *S. haemoltyticus* (n = 23; 100%) of MS_B_ phenotype, gene *msrA* was present.

**Figure 5:**
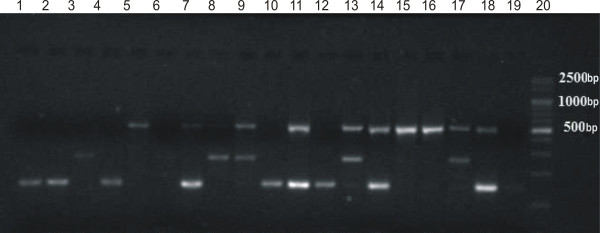
**An example of the outcome of the multiplex PCR reaction for determining the genes coding aminoglycoside resistance *aac(6′)/aph(2″) *(491 bp), *aph(3′)-IIIa *(292 bp), *ant(4′)-Ia *(135 bp).** Legend: lanes 1 – 16 studied samples; 17 – positive control reference *aac(6')/aph(2")* and *aph(3')-IIIa* W2/60; 18 – positive control reference *aac(6')/aph(2")* and *ant(4')-Ia* W2/9, 19 – negative control, 20 – marker (Eurx Perfect TM 100 bp DNA Ladder).

**Figure 6:**
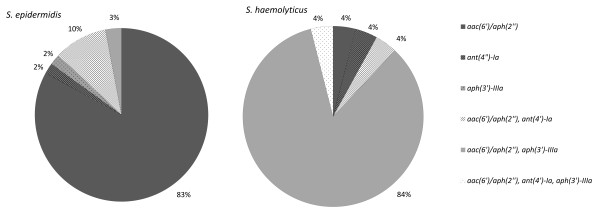
**Percentage of aminoglycoside resistance genes: *aac(6′)/aph(2″), aph(3′)-IIIa, ant(4′)-Ia *** **in isolates of the species *S. epidermidis *** **and *S. haemolyticus.***

Among the genes coding aminoglycoside resistance, *aac(6′)/aph(2″)* gene was the most frequent, and the genes more rare were *aph(3′)-Ia* and *aph(3′)-IIIa*. In *S. epidermidis*, isolates with the *aac(6′)/aph(2″)* gene were prevalent (n = 49; 83%), while in *S. haemolyticus*, the *aac(6′)/aph(2″)* was the most frequent together with *aph(3′)-IIIa* (n = 22; 84%) (Figures 5 and 6).

## Discussion

Infections caused by coagulase-negative staphylococci are among the most common causes of death among infants with very low birth weight [14]. This is due to the fact that staphylococcal biofilm can be formed on biomaterials, from which are made various types of devices used in patients chronically hospitalized. Bacteria growing in biofilm are characterized by an increased resistance to the host immune system and antibiotic use, making it more difficult and significantly extending the patient’s hospitalization [1,15].

The researched CNS isolates demonstrated multi-drug resistance. The studied *S. epidermidis* isolates and *S. haemolyticus* were resistant to methicillin, in 98% and 100%, respectively, as well as to the majority of the remaining antibiotics that were studied. These results are concurrent with the literature data where for CNS isolated from neonatal infections the percentage of methicillin-resistant strains (methicillin-resistant coagulase-negative staphylococci, MRCNS) was in the range of 86-100%, and, moreover, these bacteria was connected with multiple resistance to other antibiotics [6,7].

Investigation of the *mecA* gene in CNS using the PCR technique is nowadays regarded as the gold standard with a view to determining methicillin resistance [11]. In our study, meticillin-resistance phenotype for all the researched strains was confirmed by the detection of the *mecA* gene. Similar results were described by other authors [11].

The group of isolates was characterized by high erythromycin resistance which was 84% for isolates of *S. epidermidis* and 100% for *S. haemolyticus*. The results obtained are similar to those of Bialkowska-Hobrzanska et al. where 100% of isolates *S. epidermidis* and 92% of *S. haemolyticus* isolated from neonates were erythromycin-resistant [16], as well as similar to the data obtained by Abd El Hafez et al. where the erythromycin resistance for isolates of *S. epidermidis* coming from neonates was 86% [6].

Clindamycin resistance was demonstrated in 26% of isolates of *S. epidermidis* and 28% of isolates of *S. haemolyticus*. The determined percentage of clindamycin resistance was significantly lower than that described by Bialkowska-Hobrzanska et al. where for *S. epidermidis* it was equal to 92%, and for *S. haemolyticus* 85% [16]; similarly, Abd El Hafez et al. demonstated 75.9% of isolates of *S. epidermidis* as resistant [6], and higher than that proven by van den Hoogen et al. equal to 15% for CNS altogether [17].

Among the *S. epidermidis* strains that were macrolide-resistant, the cMLS_B_ phenotype constituted 43% and MS_B_ 40% whereas in *S. haemolyticus* the most frequently detected MS_B_ phenotype occurred in 82% of isolates. Similar results were described by Gheradi et al. who researched CNS coming from hospital infections where 38% of isolates of *S. epidermidis* and 80% of isolates of *S. haemolyticus* had the MS_B_ phenotype and 28.5% of isolates of *S. epidermidis* had the cMLS_B_ phenotype [18]. However, Gatermann et al. clearly described a lower percentage of isolates of *S. haemolyticus* with the phenotype MS_B_ equal to 30.2% [19]. For the isolates of *S. epidermidis* researched on with the cMLS_B_ and iMLS_B_ phenotypes, the occurrence of the *ermC* gene was demonstrated and with the MS_B_ phenotype, the *msrA* gene. However, 3 out of 5 *S. haemolyticus* isolates with the cMLS_B_ phenotype had the *msrA* gene, but did not have the *emrC* gene. Moreover, in the group of isolates of *S. haemolyticus* with the MS_B_ resistance phenotype, it was demonstrated that one isolate had no *msrA* gene. Probably, those isolates had other genes coding the erythromycin resistance. The recorded lack of *ermA* and *ermB* genes among the isolates researched on is in conformity with the results of other scientists researching CNS who indicated the presence of these genes only as high as 1.8% – 7.2% for *ermA* and below 1% for *ermB*[20].

The gentamicin resistance was 93% for S*. epidermidis*, 100% for *S. haemolyticus* and it was higher than quoted in the literature for *S. epidermidis* 69.2 – 89.5% [17,21] and for CNS altogether 85% except the results obtained by Bialkowska-Hobrzanska et al. where for both *S. epidermidis* and S*. haemolyticus* the resistance equaled 100% [16]. In the case of amikacin, the resistant strains of S*. epidermidis* constituted 51% of those studied, whereas 31% of *S. haemolyticus* isolates were amikacin-resistant.

From among the genes coding aminoglycoside resistance, the most frequently identified gene was *aac(6′)/aph(2″)* which both in *S. epidermidis* and S*. haemolyticus* occurred in 90% of cases. In the case of CNS isolates isolated from neonates researched by Klingenberg et al. 69.4% of subjects had the *aac(6′)/aph(2″)* gene [21]. Also, among isolates coming from various groups of patients with hospital infections, the main role in coding aminoglycoside resistance had the *aac(6′)/aph(2″)* gene occurring in 70 – 90% of CNS isolates [13]. Characteristic of *S. haemolyticus* was the presence, along with the *aac(6′)/aph(2″)* gene, of another gene coding aminoglycoside resistance, meaning *aph(3′)-IIIa* (78%). The comparison of resistance obtained by means of phenotypic methods and genotypic methods enabled observing certain discrepancies. Similarly, Choi et al. observed that only in ca. 50% of CNS isolates there existed a correlation between the phenotype of amikacin resistance and the genotype [13]. According to recommendations of EUCAST (2012) [10], the isolates demonstrating gentamicin resistance are to be treated as having a general aminoglycoside resistance with the exception of streptomycin, as this one is most probably coded by the *aac(6′)/aph(2″)* gene the product of which modifies antibiotics such as gentamicin, kanamycin, tobramycin and amikacin [22]. The phenotype of gentamicin resistance, despite the missing *aac(6′)/aph(2″)* gene, may be caused by the presence of other genes coding aminoglycoside resistance which were not determined in the method used. The observed lack of aminoglycoside resistance phenotype in 3 isolates of *S. epidermidis* having the *aac(6′)/aph(2″)* gene probably signifies the loss of functionality of that gene.

The determination of glycopeptide resistance constitutes a significant part of the characteristics of the CNS isolates as these antibiotics are most frequently administered in the empirical treatment of MRCNS infections in VLBW neonates. In the pool of the studied CNS isolates, all of them were susceptible to vancomycin, which is concurrent with observations made by other researchers dealing with CNS isolated from neonatal infections [5,6,17]. Nevertheless, the MIC value for individual isolates achieved the point of 4 mg/L which is the cut-off point for resistant isolates [10]. The fact of obtaining vancomycin resistance by coagulase-negative staphylococci is confirmed by cases of vancomycin resistance among CNS isolates isolated from infections in various groups of patients, which were recorded by several different research groups [23]. In the case of teicoplanin in the pool of isolates researched, resistance was proven for 13% of *S. epidermidis* and 3% of S*. haemolyticus*, which constitutes a conformation of increasing glycopeptide antibiotic resistance. Similar results were obtained by Trueba et al. where 30.4% of isolates of *S. epidermidis* and 35.7% of *S. haemolyticus* turned out to be teicoplanin-resistant [24] and Kristyof et al. where 32% of *S. haemolyticus* demonstrated resistance [25].

## Conclusions

The multi-drug resistance profiles obtained for isolates of coagulase-negative staphylococci isolated from infections of VLBW neonates hospitalized in NICUs indicate that there is a need to constantly monitor the resistance of these strains. At present, the drugs of choice are vancomycin and teicoplanin with the reservation that a quick selection of teicoplanin-resistant isolates of CNS is possible.

## Competing interests

The authors declare that they have no competing interests.

## Authors’ contributions

MBW designed the study, analyzed and interpreted the data, financially supported the study and wrote the manuscript; MBK and EG collected the data about VLBW neonates; MS collected the CNS isolates; MG and ML performed the molecular studies; JWM and PBH designed the Polish Neonatology Surveillance Network (PNSN); MB prepared the literature. All authors read and approved the final manuscript.

## References

[B1] Otto M *Staphylococcus epidermidis* – the ‘accidental’ pathogen Nat Rev Microbiol 2009 12 555 567 10.1038/nrmicro2182 19609257 PMC2807625

[B2] Gheibi S Fakoor Z Karamyyar M Khashabi J Ilkhanizadeh B Farzin Asghari-Sana F Mahmoodzadeh H Majlesi A Coagulase Negative Staphylococcus; the Most Common Cause of Neonatal Septicemia in Urmia, Iran. Iran J Pediatr 2008 12 237 243

[B3] Hira V Sluijter M Estevão S Horst-Kreft D Ott A De Groot R Hermans PW Kornelisse RF Clinical and molecular epidemiologic characteristics of coagulase-negative staphylococcal bloodstream infections in intensive care neonates Pediatr Infect Dis J 2007 12 607 612 10.1097/INF.0b013e318060cc03 17596803

[B4] Dimitriou G Fouzas S Giormezis N Giannakopoulos I Tzifas S Foka A Anastassiou DE Spiliopoulou I Mantagos S Clinical and microbiological profile of persistent coagulase-negative staphylococcal bacteraemia in neonates Clin Microbiol Infect 2011 12 1684 1690 10.1111/j.1469-0691.2011.03489.x 21463392

[B5] Villari P Sarnataro C Iacuzio L Molecular Epidemiology of *Staphylococcus epidermidis* in a Neonatal Intensive Care Unit over a Three-Year Period J Clin Microbiol 2000 12 1740 1746 10790091 10.1128/jcm.38.5.1740-1746.2000 PMC86575

[B6] Abd El Hafez M Khalaf NG El Ahmady M Abd El Aziz A Hashim Ael G An outbreak of methicillin resistant *Staphylococcus epidermidis* among neonates in a hospital in Saudi Arabia J Infect Dev Ctries 2011 12 692 699 21997937 10.3855/jidc.1293

[B7] Qu Y Daley A Istivan T Garland S Deighton M Antibiotic susceptibility of coagulase -negative staphylococci isolated from very low birth weight babies: comprehensive comparisons of bacteria at different stages of biofilm formation Ann Clin Microbiol Antimicrob 2010 12 16 10.1186/1476-0711-9-16 20504376 PMC2902406

[B8] Gastmeier P Geffers C Schwab F Fitzner J Oblader M Rüden H Development of a surveillance system for nosocomial infections: the component for neonatal intensive care in Germany J Hosp Infect 2004 12 126 131 10.1016/j.jhin.2003.12.038 15183242

[B9] Wojkowska-Mach J Borszewska-Kornacka M Domanska J Gadzinowski J Gulczynska E Helwich E Kordek A Pawlik D Szczapa J Klamka J Heczko PB Early-onset infections of very-low-birth-weight infants in Polish neonatal intensive care units Pediatr Infect Dis J 2012 12 691 695 10.1097/INF.0b013e3182567b74 22466319

[B10] EUCAST - European Committee on Antimicrobial Susceptibility Testing Version 2.0, valid from 2012-01-01 2012 http://www.eucast.org/fileadmin/src/media/PDFs/EUCAST_files/Disk_test_documents/EUCAST_breakpoints_v_2.0_120101.pdf

[B11] Pereira EM Schuenck RP Malvar KL Iorio NL Matos PD Olendzki AN Oelemann WM Dos Santos KR *Staphylococcus aureus*, *Staphylococcus epidermidis* and *Staphylococcus haemolyticus*: methicillin-resistant isolates are detected directly in blood cultures by multiplex PCR Microbiol Res 2010 12 243 249 10.1016/j.micres.2009.03.003 19616418

[B12] Zmantar T Chaieb K Ben Abdallah F Ben Kahla-Nakbi A Ben Hassen A Mahdouani K Bakhrouf A Multiplex PCR detection of the antibiotic resistance genes in *Staphylococcus aureus* strains isolated from auricular infections Folia Microbiol 2008 12 35 362 10.1007/s12223-008-0005-2 18759121

[B13] Choi SM Kim SH Kim HJ Lee DG Choi JH Yoo JH Kang JH Shin WS Kang MW Multiplex PCR for the detection of genes encoding aminoglycoside modifying enzymes and methicillin resistance among *Staphylococcus* species J Korean Med Sci 2003 12 631 636 14555812 10.3346/jkms.2003.18.5.631 PMC3055104

[B14] Stoll BJ Hansen N Fanaroff AA Wright LL Carlo WA Ehrenkranz RA Lemons JA Donovan EF Stark AR Tyson JE Oh W Bauer CR Korones SB Shankaran S Laptook AR Stevenson DK Papile LA Poole WK Late-onset sepsis in very low birth weight neonates: the experience of the NICHD Neonatal Research Network Pediatrics 2002 12 285 291 10.1542/peds.110.2.285 12165580

[B15] Crossley K Jefferson K Archer G Fowler V Saphylococci In Human Disease 2009 Oxford: Wiley- Blackwell

[B16] Bialkowska-Hobrzanska H Jaskot D Hammerberg J Molecular characterization of the coagulase-negative staphylococcal surface flora of premature neonates J Gen Microbiol 1993 12 2939 2944 10.1099/00221287-139-12-2939 8126420

[B17] van den Hoogen A Gerards L Verboon-Maciolek M Fleer A Krediet T Long-term trends in the epidemiology of neonatal sepsis and antibiotic susceptibility of causative agents Neonatol 2010 12 22 28 10.1159/000226604 19571584

[B18] Gherardi G De Florio L Lorino G Fico L Dicuonzo G Macrolide resistance genotypes and phenotypes among erythromycin-resistant clinical isolates of *Staphylococcus aureus* and coagulase-negative staphylococci, Italy FEMS Immunol Med Microbiol 2009 12 62 67 10.1111/j.1574-695X.2008.00499.x 19076222

[B19] Gatermann SG Koschinski T Friedrich S Distribution and expression of macrolide resistance genes in coagulase-negative staphylococci Clin Microbiol Infect 2007 12 777 781 10.1111/j.1469-0691.2007.01749.x 17501977

[B20] Bouchami O Achour W Mekni MA Rolo J Ben HA Antibiotic resistance and molecular characterization of clinical isolates of methicillin-resistant coagulase-negative staphylococci isolated from bacteremic patients in oncohematology Folia Microbiol 2011 12 122 130 10.1007/s12223-011-0017-1 21431912

[B21] Klingenberg C Sundsfjord A Rønnestad A Mikalsen J Gaustad P Flaegstad T Phenotypic and genotypic aminoglycoside resistance in blood culture isolates of coagulase-negative staphylococci from a single neonatal intensive care unit, 1989–2000 J Antimicrob Chemother 2004 12 889 896 10.1093/jac/dkh453 15471996

[B22] Liakopoulos A Foka A Vourli S Zerva L Tsiapara F Protonotariou E Dailiana Z Economou M Papoutsidou E Koutsia-Carouzou C Anastassiou ED Diza E Zintzaras E Spiliopoulou I Petinaki E Aminoglycoside-resistant staphylococci in Greece: prevalence and resistance mechanisms Eur J Clin Microbiol Infect Dis 2011 12 701 705 10.1007/s10096-010-1132-7 21222013

[B23] Juárez-Verdayes MA Reyes-López MA Cancino-Díaz ME Muñoz-Salas S Rodríguez-Martínez S de la Serna FJ Hernández-Rodríguez CH Cancino-Díaz JC Isolation, vancomycin resistance and biofilm production of *Staphylococcus epidermidis* from patients with conjunctivitis, corneal ulcers, and endophthalmitis Rev Latinoam Microbiol 2006 12 238 246 18293657

[B24] Trueba F Garrabe E Hadef R Fabre R Didier CJ Tsvetkova K Chesneau O High prevalence of teicoplanin resistance among *Staphylococcus epidermidis* strains in a 5-year retrospective study J Clin Microbiol 2006 12 1922 1923 10.1128/JCM.44.5.1922-1923.2006 16672444 PMC1479201

[B25] Kristóf K Kocsis E Szabó D Kardos S Cser V Nagy K Hermann P Rozgonyi F Significance of methicillin-teicoplanin resistant *Staphylococcus haemolyticus* in bloodstream infections in patients of the Semmelweis University hospitals in Hungary Eur J Clin Microbiol Infect Dis 2011 12 691 699 10.1007/s10096-010-1142-5 21222010

